# Patient perspectives on advance euthanasia directives in Huntington’s disease. A qualitative interview study

**DOI:** 10.1186/s12910-022-00838-0

**Published:** 2022-10-10

**Authors:** Marina R. Ekkel, Marja F.I.A. Depla, Els M.L. Verschuur, Ruth B. Veenhuizen, Cees M.P.M. Hertogh, Bregje D. Onwuteaka-Philipsen

**Affiliations:** 1 Department of Medicine for Older People, Amsterdam UMC location Vrije Universiteit Amsterdam, De Boelelaan 1117, Amsterdam, The Netherlands; 2 Amsterdam Public Health research institute (APH), Aging & Later Life, Amsterdam, The Netherlands; 3 Huntington Expert Centre Atlant, Apeldoorn, The Netherlands; 4 Lung Alliance Netherlands, Amersfoort, The Netherlands; 5 Department of Public and Occupational Health, Amsterdam UMC location Vrije Universiteit Amsterdam, De Boelelaan 1117, Amsterdam, The Netherlands

**Keywords:** Huntington’s disease, Qualitative research, Advance euthanasia directive, Advance care planning.

## Abstract

**Background:**

Huntington’s disease (HD) has a poor prognosis. For HD patients in the Netherlands, one way of dealing with their poor prognosis is by drawing up an advance euthanasia directive (AED). Little is known about the perspectives of HD patients on their AED.

**Aim:**

To gain insight into patients’ views on and attitudes towards their AED, and changes over time.

**Methods:**

A longitudinal qualitative interview study using 1 to 6 semi-structured interviews over a period of maximum three years. Nine HD patients (5 outpatient clinic, 3 day care, 1 assisted living facility) who either had an AED or were thinking about drawing it up participated in this study.

**Results:**

We identified two themes that characterize patients’ perspectives on their AEDs: (1) general character of the AED; (2) uncertainty around their AED. Ad (1) The conditions that the participants described in their AED were generally not very specific for the person. Mostly they were general notions of unbearable suffering. Familiarity with HD in the family could play a role in drawing up an AED. Ad (2) Participants generally were aware of the tentative character of their AED and could have doubts concerning their own willingness or the willingness of others in the future. Sometimes these doubts were so great, that it prevented them from drawing up an AED. However, patients did not alter their AED during the follow-up period or changed in their view or attitude on their AED.

**Conclusion:**

HD patients that draw up an AED usually describe general conditions for euthanasia and recognize that these conditions may change as the disease progresses. An AED or the wish to draw one up may be a good conversation starter for conversations about goals and preferences for future care.

## Background

Huntington’s disease (HD) is an autosomal dominant neurodegenerative disease and is typically diagnosed around age 30–50 [1]. Patients with HD experience complex and unpredictable changes in their physical, cognitive, emotional and behavioral functioning, leading to a decline in functional capacity and loss of independence, and ultimately, death [2]. HD has a clinical course of 17–20 years until death and at present, there is no cure for HD [1].

Patients with HD face an uncertain and fearful future. Fears for the future involve fear for physical and mental deterioration, pain, increasing dependency, and fear for the loss of self [3, 4]. In the Netherlands, one way of dealing with these fears, is to make arrangements for the future, especially for euthanasia [5]. Euthanasia is defined as the active termination of life at a patient’s voluntary and well-informed request. Euthanasia or physician assisted suicide (PAS) is only possible when strict criteria are met (in line with the Dutch Euthanasia act of 2002 [6]). See Table 1 for the criteria of due care associated with euthanasia and PAS. A physician can never be obliged to honor a euthanasia request. Booij et al. [7] reported that the majority of end-of-life wishes of HD patients concerned euthanasia. Subsequently, if HD patients had an advance directive, this usually was an advance euthanasia directive (AED) [7].

**Table 1:** Criteria of due care

The statutory due care criteria say that the physician must: 1. Be satisfied that the patient’s request is voluntary and well considered. 2. Be satisfied that the patient’s suffering is unbearable, with no prospect of improvement. 3. Have informed the patient about their situation and prognosis. 4. Have come to the conclusion, together with the patient, that there is no reasonable alternative in the patient’s situation. 5. Have consulted at least one other, independent physician, who must see the patient and give a written opinion on whether the due care criteria set out above have been fulfilled. 6. Have exercised due medical care and attention in terminating the patient’s life or assisting in the patient’s suicide.	

Approximately 6% of Dutch older adults has an AED [8]. The percentage of HD patients with an AED is unknown. In 2010, the percentage of deaths by euthanasia was higher in patients with HD (12–21%) than in the general population (3%) and in patients with cancer (6%) [7, 8]. In older people, possession of an AED increases the likelihood of requesting euthanasia, but having an AED does not necessarily mean that people will request euthanasia [9].

An AED seems to be a way for Dutch HD patients to deal with their bad prognosis, as it brings them a certain prospect of a way out [10]. It is unknown how patients perceive and shape this prospect of a way out and what their expectations are of their AED. Furthermore, in what way do they make changes to their AED over time? Knowledge of the patient’s perspective on their AED can help physicians in guiding patients with regard to their end-of-life wishes. The aim of this study is to gain insight into patients’ views and attitudes on their AED and changes over time.

## Methods

### Study design

A longitudinal qualitative study, in which multiple semi-structured interviews were performed with patients with HD who received outpatient care.

### Setting and participants

This study is a continuation of a Dutch study originally involving 12 HD outpatients [5]. Participants were recruited from four Dutch nursing homes that provide specialized outpatient care to patients with HD. Inclusion criteria were: (1) being able to understand the goals of the study, (2) speaking comprehensively in Dutch, and (3) being able to give informed consent. A purposive sampling strategy was used to maximize variety in the sampling of participants by gender, age, disease stage, and family living conditions.

We aimed to follow these patients for a period of maximum three years. We interviewed participants at an interval of 6–18 months, depending on whether there had been certain life events or a decline in health status in the previous months, which was checked every 6 months. Inclusion ended due to pragmatic considerations (difficulty to include new participants and time restraints).

Euthanasia was discussed with all participants. Three participants indicated that euthanasia would not be an option for them. In the current study we excluded these participants and focused only on the nine participants who either had drawn up an AED or were intending to draw it up.

### Procedure

Nurses who were involved in outpatient care judged which patients would be suitable for participating, based on the inclusion criteria and the purposive sampling strategy. They informed the patients about the study and asked whether the researcher (ME) could approach them for inclusion. Subsequently, the researcher approached these patients and informed them about the study verbally and through an information letter. Participants gave written informed consent before the interview. The interviews were carried out face to face at a time and place convenient to the participants (usually their homes). The interviews took place between August 2017 and September 2020 and the first author (ME), a psychologist, conducted all interviews. The interviews lasted 16 to 82 min (52 min on average. Seven participants were interviewed individually but for 2 participants a health-care professional was present for multiple interviews on request of the participant. This health-care professional was asked not to contribute and any comments she made were not used for the data analysis.

### Data collection

A topic list was formulated that was used as a guide during the interviews (see [5]). The interviews focused on (1) thoughts and attitudes towards the future, future care and the end of life, and (2) discussing these topics with family members and health-care professionals. The subsequent interviews mainly consisted of the same questions, but also focused on changes in health status, living circumstances, etc., and its relation to views on the future. Field notes were kept, describing reflections on the interviews. The interviews were audio-recoded, transcribed verbatim, and all personal identifiers were removed.

### Data analysis

The transcripts were read and re-read by ME and MD in order to become familiar with the data. ME and MD independently coded the transcripts. These codes were compared and discussed in several research meetings until agreement was reached. In order to gain insight in views on and attitudes towards AEDs and in changes over time we analyzed the interviews per patient longitudinally as case studies. Then, we did a cross-sectional analysis in which we looked at all interviews conducted. Analyses were done by ME and MD and discussed with the other members of the research team (BOP, RV, EV, and CH). Consensus was reached on all themes.

### Ethical consideration

All participants provided written informed consent before the first interview. It was emphasized that patients’ opinions would be dealt with confidentially and would not be provided to the physician or other health-care professionals. The Medical Ethics Committee of the VU University Medical Center reviewed this study protocol and concluded that the Medical Research Involving Human Subject Act (WMO) did not apply to this study. Therefore, an official approval of this study by the committee was not required (VUmc METc 2017.218).

## Results

### Study sample

The demographic details and the number of interviews are shown in Table 2. One participant was interviewed once, two participants were interviewed three times, three participants were interviewed four times, two participants five times and one participant was interviewed six times.

**Table 2:** Demographic details of the participants

Demographics	Participants (n = 9)
Female (n, %)	6 (67%)
Age in years (mean, range)	52 (40 to 68)
Married or living with partner (n, %)	6 (67%)
Number of children (mean, range)	2.1 (0 to 6)
Health care use (n, %)	
Outpatient clinic	5 (56%)
Day care and outpatient clinic	3 (33%)
Assisted living facility and outpatient clinic	1 (11%)
Familiar with HD before they were diagnosed (n, %)	6 (67%)
Chose to undergo predictive genetic testing (n, %)	4 (44%)
Time since predictive genetic testing (according to patient) in years (mean, range)	14 (10 to 25)
Diagnosed with HD (according to patient) (n, %)*	8 (89%)
Time since diagnosis (according to patient) in years (mean, range)	6.8 (0.2 to 15)
Number of interviews (total, range)	35 (1 to 6)
Duration in minutes (mean, range)	52 (16 to 82)
Euthanasia (in first interview)	
Drawn up an AED	6 (67%)
Thinking about drawing up an AED	3 (33%)

*One patient claimed that she did not have HD yet and experienced no symptoms, even though she received HD-related care for several years

All participants of the current study (n = 9) brought up the topic of euthanasia themselves. Five participants started talking about euthanasia when the opening question (“*Do you ever think about the future?*”) was asked by the interviewer. Two participants took the initiative in talking about euthanasia later on in the interview, and two even before the opening question was asked.

### Themes

We identified two themes which reflected the different ways in which the participants spoke about their AED: (1) General character of the AED and (2) Uncertainty around their AED.

#### 1. General character of the AED

The participants differed in the extent to which their AED was specific for them as a person. There was one participant (P6) that had a detailed statement based on what made life worth living specifically for her. She defined what gave her life meaning and derived from this when life would have become meaningless for her.

*P6.1: “I am always creative and working with my hands. Suppose that at some point I can’t do that anymore, that could also be a consideration for me, […], to say I’m done. […] Dementia. That’s another thing that makes me think: thanks but no thanks [laughs]. […] Look, if, for example, my mind is still clear, you know, and I can still use my hands, but I would, for example, be fed through a feeding tube, then that wouldn’t be a big problem at all. […] No, if that meant* *I could function OK, then I think: fine. […] Look, even if it meant I wouldn’t be able to talk very well, but I could use a computer or something, well, fine. Look, I’d still be able to communicate. But if I can’t do that anymore, so not be able to convey to others what I want, what I mean, and not be able to express myself, I would really hate that.”*

Most participants, however, indicated which conditions they no longer wanted to experience, but they did so in such general terms that it could hardly be traced back to specific circumstances or activities that have made life meaningful for them until now. Frequently mentioned issues were: becoming a vegetable, needing a wheelchair, having to go to a nursing home, getting dementia, having an empty look, being dependent on others, and being a burden to others.

*P3.2: “That I’m going to end up like a vegetable, that’s not going to happen. And that’s why I have my AED ready.”*

*P8.1: “Getting dementia. […]. If that happens, then I’m going to stop. I’ve already arranged everything.“*.

*P9.3: “Well, I still don’t want to go to a nursing home. So if I can’t live here anymore, well, then I’ll just quit.”*

There was one participant (P12) who, even though she shared in the interview that she did not want to end up in a nursing home, did not even include this statement in her AED. She downloaded her AED from the internet and did not add any personal remark to it. Therefore it could be applicable to almost everyone.

*P12.1: “I got it [AED] somewhere online because it’s hard for me to draw that up. So I got it off the internet.”*

So, in most participants we did not see a very person-specific interpretation of the AED.

*References to conditions of family members*.

Participants also differed in the extent to which they were guided by examples of family members with HD in drawing up their AED. For some participants, these experiences played a major role in formulating the conditions for euthanasia. Participants stated that they do not want to experience certain things that they have seen with family members.

*P4.1: “In the end my mother was only sleeping, […]. That was difficult. Yes, so then we said, if we become that ill, let it be over soon. We don’t have to become that old with it. Not as old as mother, no.”*

*P11.1: “At one moment my brother was in a wheelchair and all kinds of things. Then I think: well, I can’t bear the thought of that right now. Yes, you don’t want that.“*

*P12.1: “Because I don’t want that ending. Like my mother, who was ill. I don’t want that.“*

#### 2. Uncertainty around their AED

Participants differed in the extent to which they were uncertain about their AED and were aware that their AED could change over time. Most participants were aware that their AED, as they had described it, might not be carried out.

The first aspect of their doubts concerned participants’ *own willingness* to initiate the euthanasia request in accordance with their AED. At the time of the interview, the participants thought they knew at what moment they no longer wanted to live, but at the same time they realized that things could come in their path that might make them longer attached to life.

*P4.1: “That’s my limit. Then my life may be over, yes. […] My wife sometimes says: suppose you’re still very happy with the grandchildren. Well, then I’ll wait for a while, but I wouldn’t say it will take much longer. I don’t think so. We shouldn’t be too rigid about that either, but then again, if my life isn’t fun anymore, it’s over.”*

*P6.5*: “*That would be a reason to consider: do I want to continue living or not. […] It could very*.

*well be that I have found other things, another hobby or whatever, that I would think: I want to continue for 10 years. I can’t say that ahead of time.”*

One participant (P7) was so aware that the AED could change over time, that she was unable to draw up her AED.

*P7.1: “Now I have a fairly full life. Not as full as before, by no means, but I’m very happy with it. And the less full it is or the less you can do, if there are just two things I like, say going outside or something like that once a day, or whatever, seeing your kids. I find it very difficult to say which moment. I don’t believe I can already.”*

Another expression of doubt about one’s *own willingness* was the question: will I be *able* to request euthanasia when the time has come? Participants realized that it would be very difficult to request euthanasia when they would see aspects of their life that would make them still attached to their life, even though the conditions that they no longer wanted to endure were present. They dealt with questions such as: How can I resist the temptation to put it off? Or: How can I force myself to get out in time? Such as P8, who explained that she had to be confronted with other patients to be reminded that she did not want that situation, and P9, who indicated that he could imagine postponing the moment of euthanasia for his daughter.

*P8.1: “I also think I have to be faced with the facts regularly to see how sick they are and what you don’t want for yourself, so to speak. I think it’s a good thing that every now and then you see people who are further along than I am, because well, then I’ll know for sure: that is really not what I want.”*

*P9.4: “For my daughter, I feel a lot of sadness, that I have to miss her. […] That she just won’t have a dad anymore, at that age already. […]. Then I would rather take a little bit longer for her sake, […], for her to find closure as good as possible. […]. Look, if she hadn’t been there, I might have already done euthanasia or something.”*

The second aspect that participants could have doubts about was the *willingness of others* to cooperate in their euthanasia request. These doubts could concern the willingness of a physician or a relative. Not all participants had spoken to their physician about their AED and when they had, it differed whether they only did so when drawing up the AED, or if they discussed it more than once. One participant (P9) initially seemed worried that his future euthanasia request would not be carried out if he had not discussed his AED frequently enough with his general practitioner. In a following interview that doubt no longer existed.

*P9.2:* *“Well, I also had my AED updated. Because of course I have to have conversations with my new GP, the Supreme Court said so. […]. There has been a ruling about euthanasia cases where people have declared in the past, if I get Parkinson’s or become demented, then I want would like to put an end to it, […]. Then you still really have to discuss this with your GP in the meantime. […] In the city I previously lived in, I had agreed with the GP that we would sit together every four, five months.”*

*P9.3: “In the city I previously lived in, I had a good GP with whom I had a conversation every 3, 4 months, […]. And then you automatically have confidence in how that will turn out [laughs]. And now, I live here over a year and a half and I have that feeling a bit less. My GP doesn’t really want to meet every 3, 4 months. […]. I just went to her a few more times, of my own accord. Because, of course, I thought: well, I’m going to make sure I follow the rules, myself. So, 2, 3 times or so, I just made another appointment.”*

*P9.4: “I just wanted to talk with my GP every once in a while, to confirm that I still have HD and still want to die, to put it plainly [laughs]. But she just made it very clear to me, that she supports it 100%. That those kinds of conversations just aren’t necessary. […]. Because euthanasia legislation also has become more flexible. […] As long as you have an AED, you can get euthanasia.”*

Another participant (P11) worried that in due course she would be dependent on the willingness of her relatives to have the AED carried out.

*P11.1: “I remember that when my brother was here in the nursing home, my mother said to the doctor: you know what’s in the advance directive, don’t you. And the doctor replied with yes. But then at one point in time he got morphine and died within a few days. […] But, look, my husband says, he sees my advance directive of course, […] and he also says: yes, we just have to see how it goes.”*

There were two participants (P3 and P12) who had no doubts whatsoever about the tentative character of the AED. Their AED was no longer under discussion and only had to be confirmed annually with their physician or kept on the shelf.

P3.3:*“The AED is there and when the time comes it will be granted. I have not discussed it with a doctor, nothing at all.”*

### Changes in the course of the interviews (longitudinal results)

So, in most participants we saw some awareness of the tentative character of their AED. However, participants did not alter their AED during the follow-up period or changed in their view or attitude on their AED. Even P4, the only participant that reached the previously mentioned boundary of needing help in taking a shower, did not mention that he altered his AED in the process.

*P4.2: “I also have an AED. If I’m no longer able to shave and brush my teeth, things like that, then I will quit life. I don’t want any help from home care or anything. […]. Becoming dependent on others, I don’t think so. […]. I’ve been a professional caregiver myself, so I could do all those things. […]. No, that doesn’t seem nice to me.”*

*P4.6: “I receive home care three times a week, and I like it. Yes, they take good care of me. […]*

### I: and what is it like for you, to receive home care?

*P: Yes, I think that’s fine. I’ve worked in healthcare myself, so I’ve done it quite often, so I don’t mind. […].*

*I: I can remember from the previous times, you were not so positive about help with showering, for example.*

### P: yes, I have completely turned around.”

## Discussion

This study set out with the aim of gaining insight into patients’ views and attitudes on their AED. Two themes were identified through which these views and attitudes could be described. First, the general character of the AED. The AEDs mostly contained general, non person-specific notions of unbearable suffering. Participants could be guided by examples of family members with HD in drawing up their AED. Second, the uncertainty around their AED, ranging from having no doubt at all whether the AED would be carried out to so much doubt that the AED did not get drawn up. Most participants were aware of the tentative character of their AED. However, participants did not alter their AED during the follow-up period, even when they had reached their described boundaries.

### General character of the AED

Euthanasia seemed an important topic for the participants, as they generally talked about it a lot in the interviews. Yet, we found that the AEDs mostly contained general, non person-specific notions of unbearable suffering. The boundaries for euthanasia were not described in a very person-specific way by participants, even when further probed by the interviewer. A previous qualitative study with patients with HD, showed that patients can have with general ideas about their wishes and that these wishes can be a vague indication of what they would not want [11]. Reasons for opting for euthanasia and drawing up an advance directive are to prevent suffering, to prevent unnecessary lengthening of life or treatment, and because of anticipatory fears of losses and multidimensional suffering [12, 13]. A possible explanation that boundaries for euthanasia are described in a general manner could be that the future is difficult to anticipate for patients. As a consequence, it is difficult to become specific when thinking about a situation that may lie years ahead. Patients possibly are mainly looking for assurance that they do not have to endure the disease until the very end.

When it comes to carrying out a euthanasia request, what are the possible consequences of not having a personal or specific AED? As long as patients are able to communicate their wishes, an AED is not required for euthanasia. However, in the euthanasia code of practice it is described that the physician may interpret the AED as the euthanasia request, if a situation arises in which the patient is no longer able to express his will. This means that, in that case, the AED has the same status as an oral request for euthanasia and the content of the AED becomes of great importance. As stated in the Code of Practice of the Regional Euthanasia Review Committees (2018), the AED must be clear and must be unmistakably applicable to the situation that has arisen [14]. In that way, it can be determined whether the current situation of the patient corresponds to the situation described in the AED. A personally prepared statement from the patients (described in one’s own words) usually has more meaning than a pre-printed form. For example, case 2017 − 103, which was an actual euthanasia case judged by the RTE, describes an AED in which was written down that the patient wanted euthanasia when she had to be admitted to a nursing home [15]. However, the committee described that this AED was rather brief as far as the description of suffering is concerned. As such, the general notion of unbearable suffering (nursing home admittance) was insufficient to assume unbearable suffering both during the disease process and just before the euthanasia was performed. A study showed that the AEDs of patients with dementia contained statements that could make their implementation difficult, and that these statements should be more specific and realistic [16].

We saw that for some participants experiences with family members with HD played a major role in drawing up an AED. Prior studies, in patients with HD and in patients with dementia, have noted the importance of experiences in the direct environment (family or friends) in drawing up an AED [11, 13, 16]. However, this experience was not a prerequisite for drawing up an AED, and in the current study not all participants had experience with HD in their family. The threatening image that these participants referred to were images of patients they had seen on the internet or in the waiting room of the hospital or outpatient clinic.

### Uncertainty around their AED

In the current study it was found that most participants were aware of the tentative character of their AED, but that there was variation between participants. When this uncertainty becomes greater, it can become more difficult to draw up an AED. These results are consistent with previous studies, in which some patients experienced difficulty in determining their limit and mentioned that their views might change [10], or saw that in the course of their illness some boundaries had shifted, although other boundaries were less likely to change [13]. Reasons for having doubts in the current study could concern the willingness of others (the physician or a relative) to cooperate in their euthanasia request. Concerning the cooperation of the physician, previous studies have shown that negative euthanasia experiences in the family cause concern when it comes to patient’s own euthanasia wishes, or cause patients to not communicate with their physician about their own wishes at all [10, 17]. This is in line with our finding that one of the participants explained that she thought it wasn’t going to be easy to get her wish for euthanasia carried out, because that is what she experienced with a family member.

Future research focusing on the perspective of physicians in dealing with (advance) euthanasia requests in HD patients will need to be undertaken to gain a full picture of the patient-physician relationship when it comes to euthanasia. Furthermore, in the current study, one participant expressed worries that in due course she would be dependent on the willingness of her relatives to have the AED carried out. In practice, however, even though previously speaking about wishes with relatives is important in a euthanasia request, relatives only have a minimal role in carrying out the euthanasia [14].

### Changes in the course of the interviews (longitudinal results)

There was one participant in the current study that adjusted his limits when it came to accepting home care. This participant acknowledged pushing his boundaries but did not show awareness that this might influence his AED. The AED was not adjusted in that case. However, this was the only participant that actually came in the situation that was previously described as being a condition for euthanasia. From other studies we know that shifting boundaries can lead to adjustments in AEDs [13], albeit that changes in preferences and related changes in formulation of advance directives were found to be relatively rare over a 6-year period [18]. A study that specifically focused on whishes concerning euthanasia found that of patients with an AED, 87% remained stable in their desire for euthanasia months before their death and that 47% eventually requested euthanasia [9]. Since these studies were not conducted with patients with HD, future studies on stability of wishes in HD patients are therefore recommended.

### Euthanasia as an element of advance care planning

Previously it was mentioned that the content of an AED is relevant when patients are no longer able to express their will. Next to the content of the AED, there are other aspects that are of importance in reviewing a request for euthanasia. Communication about wishes at the time the patient was competent is important, especially conversations with a physician [14]. Regular conversations between the patient and a physician about the AED and the possible changes in whishes further ensures the reliability of the AED [14]. It is questionable whether patients are aware of the importance of these conversations, given that not all participants in the current study had discussed their AED with their physician or did so only once, when drawing up the AED.

Next to the importance of communication when the patient is no longer competent, there are other reasons for encouraging conversations about euthanasia. First, the uncertainty around their AED. Patients are aware that their wishes can change over time. Second, having certain fears for the future and having a threatening image that patients do not want to endure may function as a good conversation starter for conversations about goals and preferences for future care. Talking about the possibilities and limitations of future care can contribute to making an informed decision by the patient.

A process that focuses on end of life wishes is advance care planning (ACP). ACP revolves around enabling individuals to define goals and preferences for future medical treatment and care, to discuss these goals and preferences with family and health-care providers and to record and review these preferences if appropriate [19]. Given this definition, it can be discussed whether talking about preferences concerning euthanasia can be seen as an element of ACP or not. However, regardless of whether discussing euthanasia can be considered an element of ACP, communication about euthanasia and an AED should be encouraged given the previously mentioned reasons.

### Strengths and limitations

A strength of this study is that it was possible to explore the perspectives of the patients by conducting face-to-face interviews. Furthermore, because of the longitudinal character, we were able to follow these patients for up to three years which can yield more robust data.

A limitation of this study is that only one of the patients was followed long enough to reach the situation mentioned in the AED. If more participants would have reached this situation, we would have gained more insight in the process of reaching a certain condition for euthanasia and possibly pushing boundaries. Even though following the participants for a period of three years can already be seen as a strength, we would recommend following patients for an even longer time period. Another limitation of this study is that we listened to what participants had to say about their AED, but we saw the actual document of only one participant. There could be a discrepancy between what patients say about their AED and what the AED contains, for example due to cognitive impairment. This study is furthermore possibly limited by the limited number of interviews, which may have resulted in insufficient saturation. Finally, selection bias may have occurred due to inclusion taking place only in outpatient nursing homes and not in other outpatient facilities or focusing on patients that do not visit any of these facilities at all. This may cause the results to not be generalizable to these patients.

## Conclusion

This interview study has provided valuable insight into the perspectives of HD patients on their AED. Patients that draw up an AED usually describe general conditions for euthanasia and recognize that these conditions may change as the disease progresses. Conversations regarding euthanasia should be encouraged and an AED or the wish to draw up an AED may be a good conversation starter for conversations about goals and preferences for future care. In this paper, several ethical issues regarding AEDs in HD were discussed: stability of preferences, level of detail in AEDs, admissibility of cited reasons and motivations, fear for anticipated suffering vs. actual suffering, competence issues, and uncertainties for professionals in interpreting wishes and requests.

## Data Availability

The transcripts of the interviews are not publicly available due to privacy concerns but are available from the corresponding author on reasonable request.

## List of abbreviations

ACP: Advance care planning.
AED: Advance euthanasia directive.
HD: Huntington’s disease.
PAS: Physician assisted suicide.
